# Emotions Are Folk Concepts in a Predicting Brain

**DOI:** 10.3390/brainsci16060614

**Published:** 2026-06-06

**Authors:** Kristen A. Lindquist

**Affiliations:** Department of Psychology, The Ohio State University, Columbus, OH 43210, USA; lindquist.83@osu.edu

**Keywords:** emotion, constructionism, allostasis, culture, cultural evolution

## Abstract

**Highlights:**

**What are the main findings?**

- This perspective provides a definition of “emotions” as folk concepts that are used by a predictive brain, drawing on models of predictive brain function.
- Emotion concepts are used to predict adaptive actions, and in turn feelings.

**What are the implications of the main findings?**

- Predictive processing itself might give rise to feelings of valence and arousal. Emotional predictions using folk concepts such as “anger” or “fear” might be a particularly useful class of predictions for navigating humans’ social worlds.

**Abstract:**

The question “what is an emotion” is as old as the study of the mind and brain. Scientists from Western, English-speaking, Industrialized, Rich, and Democratic (WEIRD) nations tend to adopt certain definitions, but these definitions do not map onto the lived experiences of all people around the world. My approach to the question “what is an emotion?” is thus to accept that emotions are folk concepts that do not “cut nature at its joints.” Instead, I draw from models of predictive brain function to ask, “how does the brain make mental states in general, and experiences that we call “emotions” in particular?” I discuss recent models of predictive processing and how it is thought that the brain gives rise to feelings of valence and, arousal, and how, in some cultures, categorical emotional predictions (e.g., “anger”) might have evolved as a particularly useful class of predictions for navigating humans’ social worlds.

## 1. Introduction

In psychology, neuroscience, philosophy, and associated domains of study, emotions are often defined as internal, subjective mental states. These subjective mental states are said to have features such as felt intensity, valence, and arousal, and to reflect a person’s abstract goals (to avoid threat, offense, loss, contamination; to gain social connection or rewards). Once triggered, emotions can be lasting and can elicit associated cognitions and actions (or appraisals/action tendencies) that shape perception and social behavior. As a speaker of English raised and living in the United States, that definition fits well with my lived experiences of emotions. Yet the Hadza of Tanzania, who speak Hadzane and live in small-scale groups in non-industrialized East Africa do not describe emotions like this at all (see [1]; for additional examples in other non-industrialized societies, see [2,3]). According to the Hadza, an emotion has features such as somatic experiences of bodily pain, tension, and temperature changes. Emotions reflect not abstract goals, but immediate situated needs (e.g., avoiding an elephant [1]). Emotions are fleeting and do not themselves cause perceptions or social behaviors—cognitions and behaviors are seen as a product of the situation, not the emotion. The conflict between these two definitions is clearly a problem for my WEIRD (Western, English, Industrialized, Rich, and Democratic; [4]) definition of emotion. Yet the problem does not end here. If an emotion is characterized by features such as felt intensity, valence, arousal, and associated cognitions about and behaviors toward the world, then is a *perception* of a beautiful vase of flowers truly distinct from an *emotion*? Is a *decision* to invest my money in a less risky index fund truly distinct from an *emotion*?

## 2. Defining Emotions

As a psychologist and neuroscientist who studies emotions, my approach to defining emotions is to accept that emotion categories such as “anger,” “disgust,” “fear,” “sadness,” “joy,” “guilt,” “pride”—whatever labels are oft applied in WEIRD scientific works—are folk categories that are unlikely to “cut nature at its joints” ([5], (265e) [6]). They name features of mental life that are carved up differently by people from different geographies, histories, and cultures. My colleagues’ and my approach instead has been to try to understand how the brain creates mental life, more generally (see [7]). In doing so, we hope to gain insight into how the brain creates the events that English speakers call “emotions,” more specifically.

When you look deeper into research on brain evolution, it becomes clear that the brain did not evolve for the purpose of creating “emotions” or even “cognitions” (see [8,9]). Rather, it likely evolved because of pressures related to metabolic regulation of the body [8,9,10,11]. As early single-celled organisms became more complex and developed the ability to move, this adaptation placed additional pressures on energy regulation because movement is energetically costly (see [9,10] for discussions). Fast forward a few billion years, and a central processing organ was an efficient way of regulating the metabolic needs of complex bodies that could move, forage for food, avoid threats in the environment, and optimize reproduction [9,10,11]. However, brains are themselves metabolically costly organs that take up a large percentage of the body’s glucose; thus, selection pressures likely favored metabolically efficient brain functions over inefficient ones (see [11] for a discussion). 

Modern psychology and neuroscience has assumed for many years that brains are reactive: waiting for events in the external world to perturb them, only at which point they engage in activity to initiate behaviors, emotions, cognitions, etc. Yet brains do not work this way. The brain achieves the efficient regulation of the body and navigates throughout the ever-changing world by continuously running an internal model—a set of best guesses—that represents the state of the body in the world [8,12,13,14,15]. This model involves representing the predicted state of the body, the predicted state of the world, and predictively adjusting physiological states and actions to meet the body’s needs [8,11,12,13,14,16]. A prediction is the brain’s expectations about the meaning of incoming sensory information, whether from inside the body or the world outside it. The predictive regulation of the body is called allostasis, and allostasis (versus reactive regulation) is both adaptive and metabolically efficient, allowing the brain to anticipate events and respond before they occur [14]. For instance, a predictive brain raises blood pressure before the body stands up to ensure that enough oxygen reaches it amidst gravitational shifts in blood flow. A predictive brain signals the need for more glucose or water well before bodily stores become critically low. A predictive brain ducks to avoid the perception of a low branch well before the head encounters it. A predictive brain with the right “hardware” (e.g., expanded frontal cortices with loosely packed neurons that afford dimensionality reduction [17]) can even predict more abstract phenomena such as how future events might unfold, or the likely behaviors and mental states of conspecifics, and can adjust its own behaviors and mental states accordingly.

Mental states in general—and emotions, thoughts, perceptions, memories, etc., in particular—are thought to be a by-product of such more abstract predictions [8,15,18,19]. Such mental states have surely themselves been adaptive in humans’ social niches over the course of phylogeny [20]. In this allostatic view of emotions, emotions are predictions produced through the filter of each person’s individual predictive model (see [7]). Predictive models are built over ontogeny, and part of the efficiency of a predictive brain relates to the fact that it only updates predictions if they are sufficiently incongruent with incoming sensory signals from the body (interoception) and world (exteroception). Prediction-incongruent sensory signals are called “prediction errors,” and brain function can thus be said to be a product of predictions that are held “in check” by prediction errors [12,13]. Prediction error causes the model to update (i.e., learning occurs) and may also initiate action to proactively address the source of error (e.g., active inference [21]). The updating of predictions over time is a mechanism via which a person’s individual, social, and cultural environments can produce idiographic and cultural variation in the person’s predictive model.

One byproduct of prediction may be feelings that have qualities of arousal and valence (see [7,8]). Predicted somatovisceral and somatomotor actions are associated with subjective arousal (see [22,23]), as is the neuronal signaling associated with learning through prediction errors (dopamine, norepinephrine [24]). In contrast, valence is thought to emerge as a product of model fit; when an internal model fits well in the current environment, this is associated with positive valence; in contrast, when the model fits poorly (i.e., there is uncertainty), this is associated with negative valence (see [25]). Arousal and valence may thus emerge as a by-product of predictive processing itself (see [7,22,23,25,26]). Collectively, arousal and valence may serve as a low-dimensional representation of the state of the body (i.e., interoception [27]) in relation to the world around it (for discussions see [7,22,23]). Perturbations to this representation, when unpredicted, can themselves serve as interoceptive prediction error that updates future predictions. For instance, interoceptive prediction errors can feed into on-going emotional experiences, such as when a person feels greater negativity following a frustrating situation because they are also subjectively hungry [28]. In contrast, people feel less negativity following a stressful speech when beta adrenergic signaling of autonomic arousal is blocked through administration of the beta blocker propranolol [29].

Critically, the human brain is also capable of incorporating more complex, abstract, and evaluative predictions about the body and the self in relation to the current environment. For instance, the brain can predict that foods are delicious, that tall cliffs are dangerous, and that losing resources would be detrimental to survival. It can also predict that reuniting with a loved one is going to be pleasant, that a competitor means one harm, and that losing a loved one will be painful. These predictions can come from past experiences, social learning, or both (see [7]). Over time, and through learning, especially via communication with other humans, these predictions can coalesce into clusters of predictions (i.e., concepts) that members of a culture share and may even label (in English) with terms such as “joy,” “fear,” “sadness” and other emotion categories [30,31].

These clusters of predictions need not be basic instincts, as categories such as “anger,” “fear,” and “sadness” are sometimes theorized to be, but are instead more akin to culturally constructed “cognitive gadgets” like language or theory of mind [32] that evolved because they conferred adaptive fitness amongst humans living in social groups. Emotion concepts were likely useful because they helped humans make predictions about self-relevant situations that tended to have occurred over time in social groups in general, but also certain cultural groups in particular. Some of those situations may be related to basic primate motivations (e.g., social affiliation; see [33]), whereas others may be quite culturally specific. For example, “anger” is a concept that has translations in many cultures, although it involves cultural-specific predictions about its associated causes, behaviors, and even physiological responses. In many WEIRD cultural groups, “anger” involves predictions related to situations where there has been an obstruction of personal goals, transgression of norms, or violation of one’s autonomy or personal property (see [34]). Correspondingly, in these contexts, behavioral predictions include asserting one’s dominance, sanctioning transgressors, and protecting one’s personal property and autonomy. It is interesting that in these contexts, anger is also associated with inflammatory reactivity [35,36], perhaps because anger commonly involves predictions involving aggressive confrontation and potential tissue damage. Yet in Japan, “anger” is associated with situations in which there is a breach to relational harmony and a desire to mend relationships and see others’ points of view [34]; it is correspondingly not associated with inflammatory reactivity [35,36], and indeed, a predictive increase in inflammation in this context would be metabolically wasteful. These examples offer ways in which the same category is stereotypically associated with different adaptive predictions across cultures.

My colleagues and I thus predict that whereas arousal and valence are likely a product of a predicting brain in general, predictions about specific discrete emotion categories (“fear,” “anger,” “disgust,” “joy,” “pride,” or whatever categories are emphasized within a social group) are likely acquired over time and in contact with others in one’s culture [7,20,37]. Emotion concepts are useful for allostasis insofar as they are efficient: they include expectations about how common situations will unfold, what they will mean for the state of the body and the self, and which behaviors are most adaptive to rectify undesirable situations and maintain desirable ones [20]. From infancy, the emotional concepts relevant to one’s culture are socialized via contact with caregivers and other humans (see [20,37,38] for discussions); social learning thus “seeds” one’s predictive model by providing concepts that anchor future learning. There is much evidence that people who possess and wield their culture’s emotion concepts in a highly precise manner (i.e., are emotion experts) have better mental, physical, and social outcomes [39]. This is likely because these individuals have well-adapted predictive models and are using those predictions successfully to navigate challenging situations, foster social bonds, make decisions, and adaptively change behavior. Consistent with this interpretation, the well-being of immigrants to a new culture improves once they have learned the emotional predictions normative to the host culture [40]—that is, once they are applying culturally appropriate predictions. If well-being stems from having a highly precise, culturally situated model, then clinical disorders with emotional symptoms at their core may be attributable to predictive models that fail to update following ill-fitting or culturally discordant predictions (see [23]).

## 3. Conclusions

In sum, my take on the question “what is an emotion” shifts the emphasis from thinking of emotions as a fixed set of categories shared by all humans across places and times, to understanding them as folk concepts that are “constructed” by a predicting brain based on the local cultural niche. This predicting brain may produce experiences of arousal and valence associated with having an embodied self [8,16,19,22], but the quality and quantity of categorical emotional predictions are deeply embedded in cultural learning [7,20]. This approach ultimately unites predictive processing models of brain function [12,13,14,16] with dual-inheritance theories of evolution that unite genetic and social inheritance as sources of human variation [20]. Within the study of emotion more specifically, this view unites constructionist theories of emotion [7,8,20] that focus on emotions as folk categories created from more basic brain operations and constitutive appraisal models of emotion that focus on the abstract meaning dimensions [41] or basic motivations [33] that can be associated with different emotion categories. This approach shifts away from debates about whether emotions are entirely “biological” or “cultural” and accepts that they are products of both biological *and* cultural evolution (and the interaction thereof) [20]. In doing so, it seeks to find both commonalities and differences in emotions across cultures that could arise from similarities or differences in genetics, ecology, social practices or the interactions of those factors [20]. This approach thus explains why some emotion concepts are common across many different cultures (as predicted in universalist approaches; e.g., [42]), whereas others are culturally bound (see [20] for a discussion). Finally, this approach explains how scientists from WEIRD backgrounds can come up with one definition of “emotion,” whereas the lived experiences of people from culturally distinct contexts might be quite different [1]. My hope is that by continuing to understand how the predictive brain functions, we can understand how it makes the mind, including those mental states we call “emotions.”

## Data Availability

No new data were created or analyzed in this study. Data sharing is not applicable to this article.
